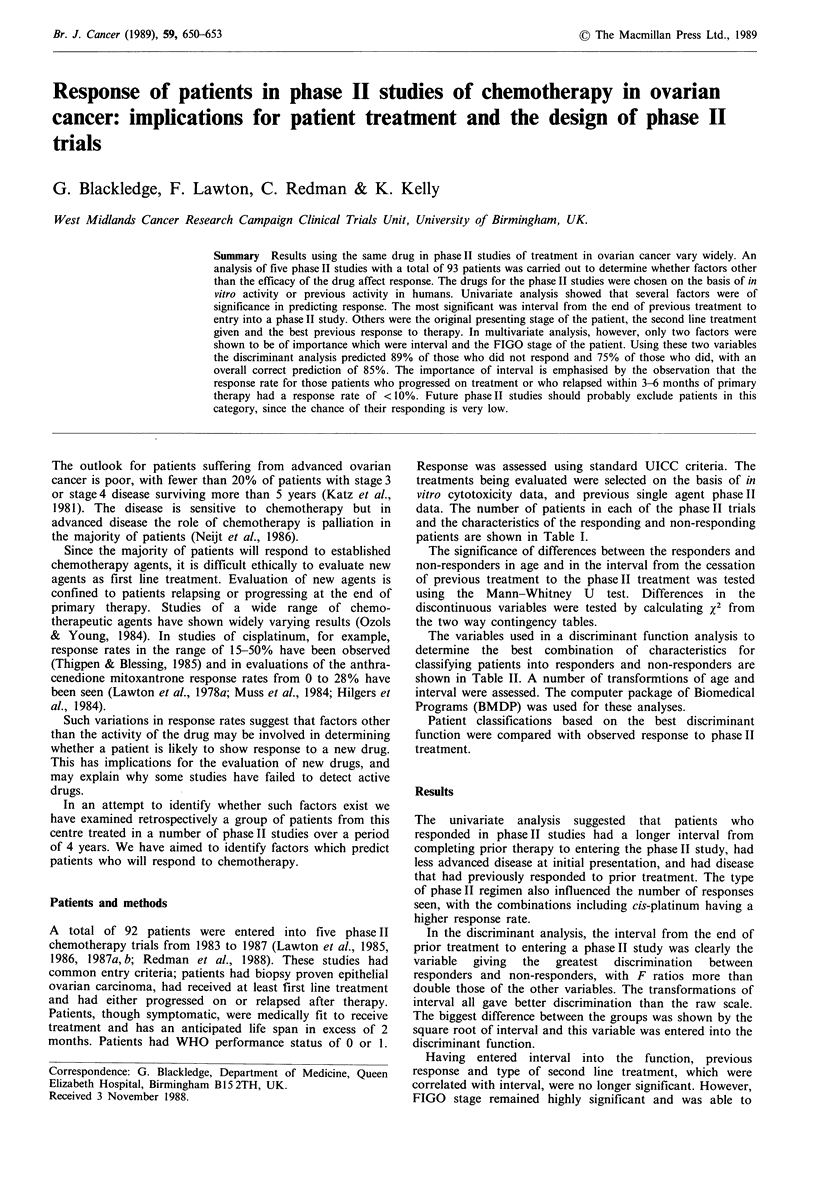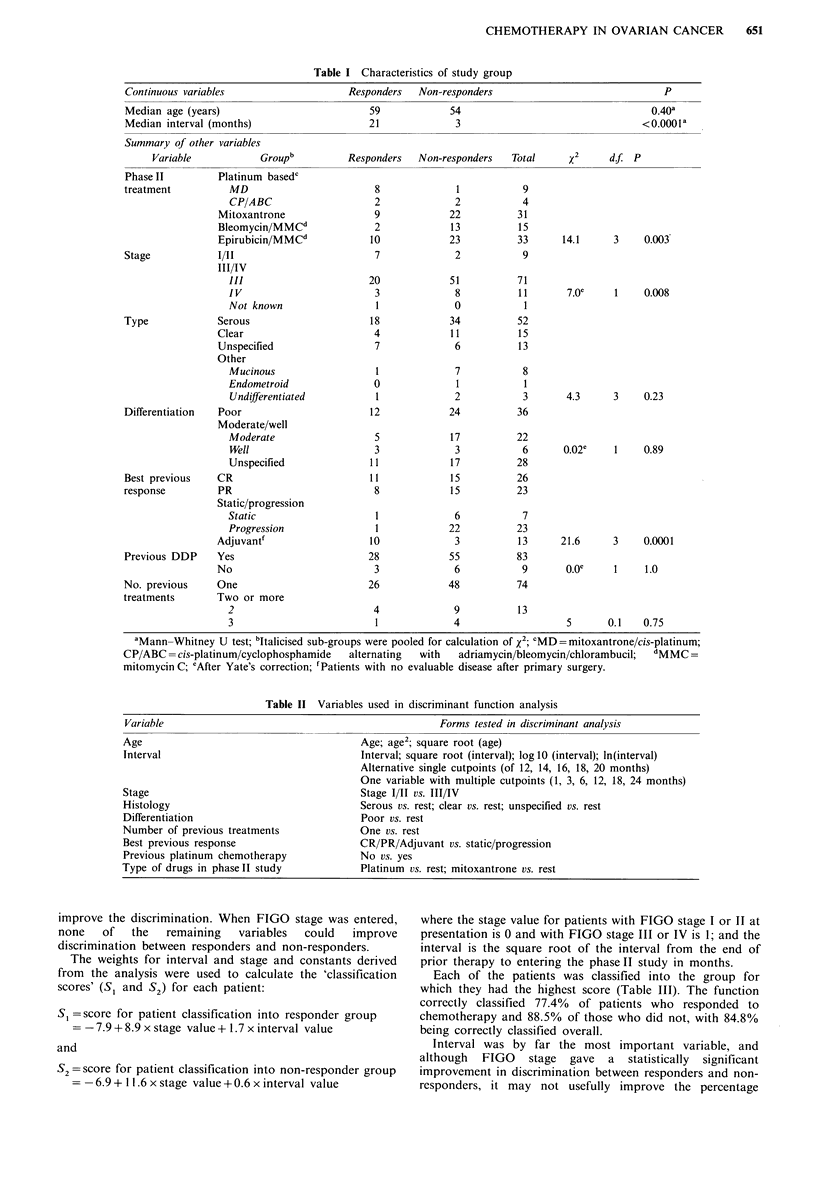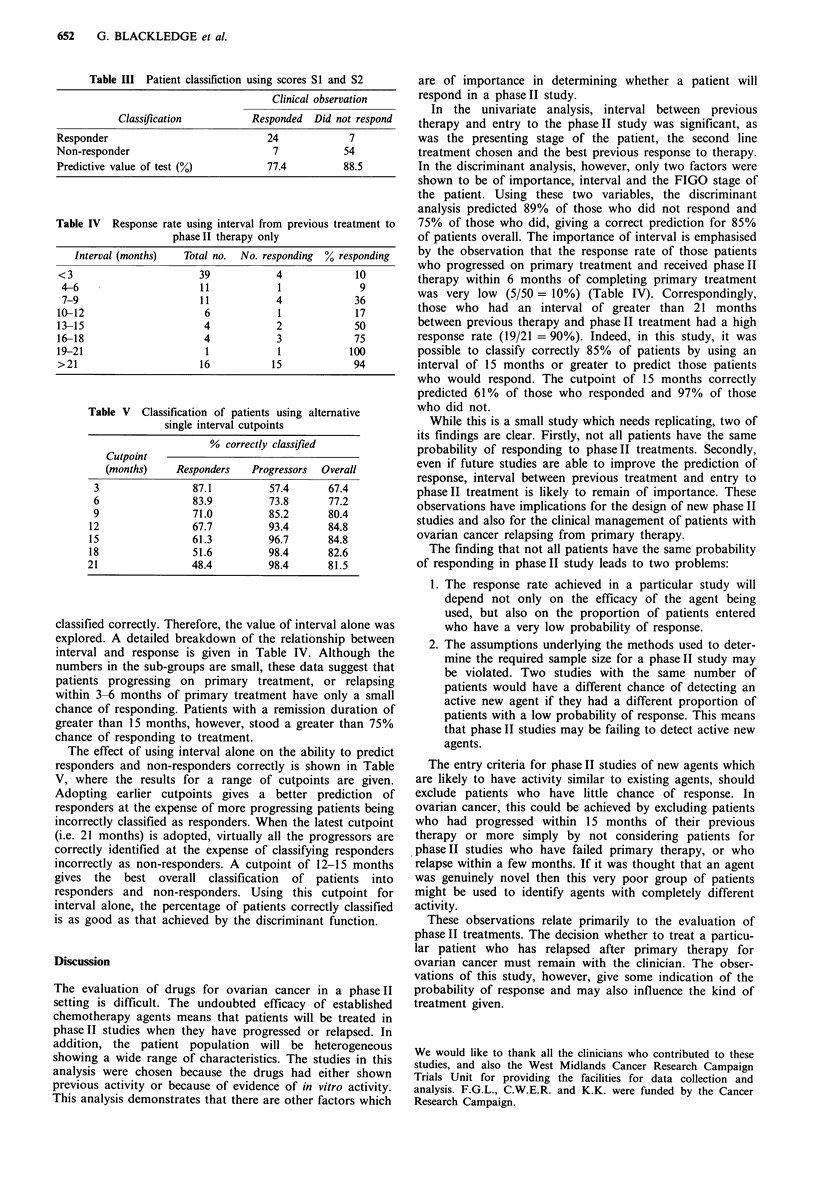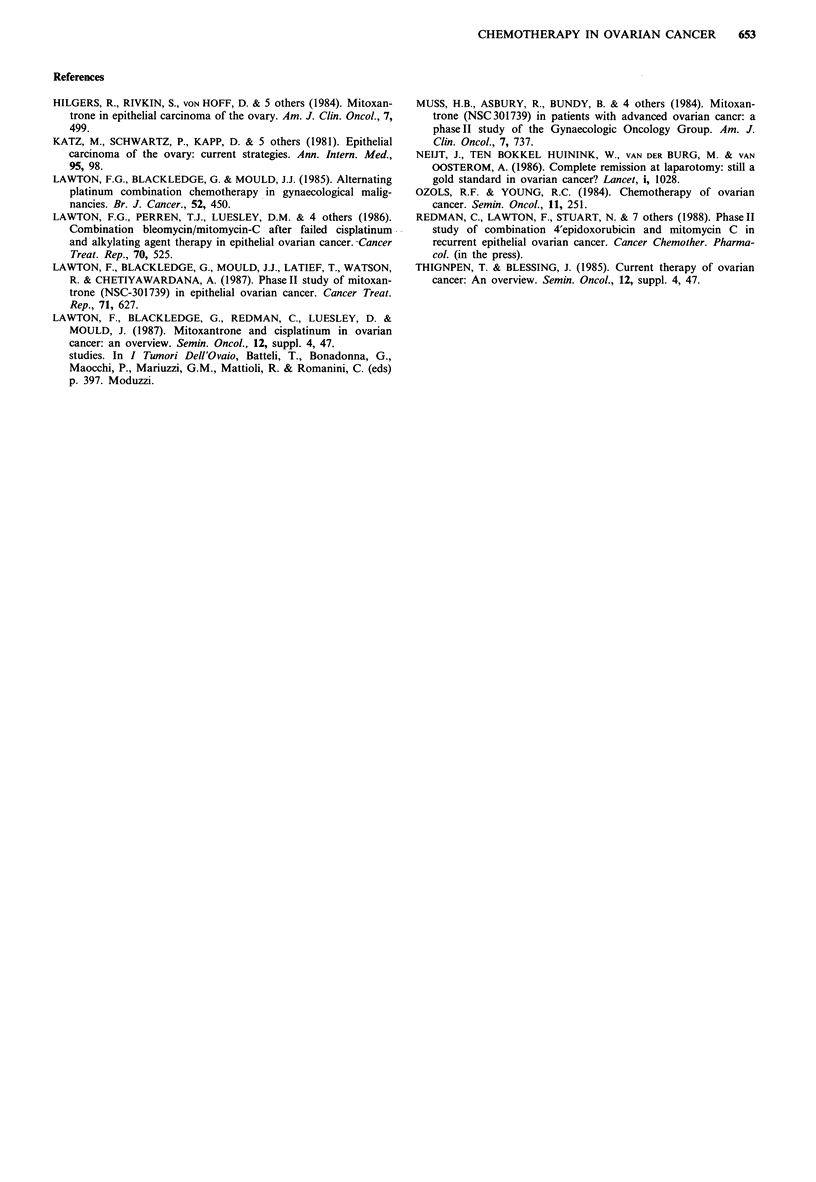# Response of patients in phase II studies of chemotherapy in ovarian cancer: implications for patient treatment and the design of phase II trials.

**DOI:** 10.1038/bjc.1989.132

**Published:** 1989-04

**Authors:** G. Blackledge, F. Lawton, C. Redman, K. Kelly

**Affiliations:** West Midlands Cancer Research Campaign Clinical Trials Unit, University of Birmingham, UK.

## Abstract

Results using the same drug in phase II studies of treatment in ovarian cancer vary widely. An analysis of five phase II studies with a total of 93 patients was carried out to determine whether factors other than the efficacy of the drug affect response. The drugs for the phase II studies were chosen on the basis of in vitro activity or previous activity in humans. Univariate analysis showed that several factors were of significance in predicting response. The most significant was interval from the end of previous treatment to entry into a phase II study. Others were the original presenting stage of the patient, the second line treatment given and the best previous response to therapy. In multivariate analysis, however, only two factors were shown to be of importance which were interval and the FIGO stage of the patient. Using these two variables the discriminant analysis predicted 89% of those who did not respond and 75% of those who did, with an overall correct prediction of 85%. The importance of interval is emphasised by the observation that the response rate for those patients who progressed on treatment or who relapsed within 3-6 months of primary therapy had a response rate of less than 10%. Future phase II studies should probably exclude patients in this category, since the chance of their responding is very low.


Br. J. Cancer (1989), 59, 650–653 © The Macmillan Press Ltd., 1989

### Response of patients in phase II studies of chemotherapy in ovarian cancer: implications for patient treatment and the design of phase II trials

G. Blackledge, F. Lawton, C. Redman & K. Kelly

*West Midlands Cancer Research Campaign Clinical Trials Unit, University of Birmingham, UK.*

**Summary** Results using the same drug in phase II studies of treatment in ovarian cancer vary widely. An analysis of five phase II studies with a total of 93 patients was carried out to determine whether factors other than the efficacy of the drug affect response. The drugs for the phase II studies were chosen on the basis of *in vitro* activity or previous activity in humans. Univariate analysis showed that several factors were of significance in predicting response. The most significant was interval from the end of previous treatment to entry into a phase II study. Others were the original presenting stage of the patient, the second line treatment given and the best previous response to therapy. In multivariate analysis, however, only two factors were shown to be of importance which were interval and the FIGO stage of the patient. Using these two variables the discriminant analysis predicted 89% of those who did not respond and 75% of those who did, with an overall correct prediction of 85%. The importance of interval is emphasised by the observation that the response rate for those patients who progressed on treatment or who relapsed within 3–6 months of primary therapy had a response rate of <10%. Future phase II studies should probably exclude patients in this category, since the chance of their responding is very low.

The outlook for patients suffering from advanced ovarian cancer is poor, with fewer than 20% of patients with stage 3 or stage 4 disease surviving more than 5 years (Katz *et al.*, 1981). The disease is sensitive to chemotherapy but in advanced disease the role of chemotherapy is palliation in the majority of patients (Neijt *et al.*, 1986).

Since the majority of patients will respond to established chemotherapy agents, it is difficult ethically to evaluate new agents as first line treatment. Evaluation of new agents is confined to patients relapsing or progressing at the end of primary therapy. Studies of a wide range of chemotherapeutic agents have shown widely varying results (Ozols & Young, 1984). In studies of cisplatinum, for example, response rates in the range of 15–50% have been observed (Thigpen & Blessing, 1985) and in evaluations of the anthracenedione mitoxantrone response rates from 0 to 28% have been seen (Lawton *et al.*, 1978*a*; Muss *et al.*, 1984; Hilgers *et al.*, 1984).

Such variations in response rates suggest that factors other than the activity of the drug may be involved in determining whether a patient is likely to show response to a new drug. This has implications for the evaluation of new drugs, and may explain why some studies have failed to detect active drugs.

In an attempt to identify whether such factors exist we have examined retrospectively a group of patients from this centre treated in a number of phase II studies over a period of 4 years. We have aimed to identify factors which predict patients who will respond to chemotherapy.

### Patients and methods

A total of 92 patients were entered into five phase II chemotherapy trials from 1983 to 1987 (Lawton *et al.*, 1985, 1986, 1987*a*, *b*; Redman *et al.*, 1988). These studies had common entry criteria; patients had biopsy proven epithelial ovarian carcinoma, had received at least first line treatment and had either progressed on or relapsed after therapy. Patients, though symptomatic, were medically fit to receive treatment and has an anticipated life span in excess of 2 months. Patients had WHO performance status of 0 or 1. Response was assessed using standard UICC criteria. The treatments being evaluated were selected on the basis of *in vitro* cytotoxicity data, and previous single agent phase II data. The number of patients in each of the phase II trials and the characteristics of the responding and non-responding patients are shown in Table I.

The significance of differences between the responders and non-responders in age and in the interval from the cessation of previous treatment to the phase II treatment was tested using the Mann–Whitney U test. Differences in the discontinuous variables were tested by calculating $\chi^2$ from the two way contingency tables.

The variables used in a discriminant function analysis to determine the best combination of characteristics for classifying patients into responders and non-responders are shown in Table II. A number of transformtions of age and interval were assessed. The computer package of Biomedical Programs (BMDP) was used for these analyses.

Patient classifications based on the best discriminant function were compared with observed response to phase II treatment.

### Results

The univariate analysis suggested that patients who responded in phase II studies had a longer interval from completing prior therapy to entering the phase II study, had less advanced disease at initial presentation, and had disease that had previously responded to prior treatment. The type of phase II regimen also influenced the number of responses seen, with the combinations including *cis*-platinum having a higher response rate.

In the discriminant analysis, the interval from the end of prior treatment to entering a phase II study was clearly the variable giving the greatest discrimination between responders and non-responders, with $F$ ratios more than double those of the other variables. The transformations of interval all gave better discrimination than the raw scale. The biggest difference between the groups was shown by the square root of interval and this variable was entered into the discriminant function.

Having entered interval into the function, previous response and type of second line treatment, which were correlated with interval, were no longer significant. However, FIGO stage remained highly significant and was able to

Correspondence: G. Blackledge, Department of Medicine, Queen Elizabeth Hospital, Birmingham B15 2TH, UK.
Received 3 November 1988.

**Table I** Characteristics of study group

| *Continuous variables* | | *Responders* | *Non-responders* | | | | *P* |
|---|---|---|---|---|---|---|---|
| Median age (years) | | 59 | 54 | | | | 0.40[a] |
| Median interval (months) | | 21 | 3 | | | | <0.0001[a] |
| *Summary of other variables* | | | | | | | |
| *Variable* | *Group*[b] | *Responders* | *Non-responders* | *Total* | $\chi^2$ | *d.f.* | *P* |
| Phase II treatment | Platinum based[c] | | | | | | |
| | *MD* | 8 | 1 | 9 | | | |
| | *CP/ABC* | 2 | 2 | 4 | | | |
| | Mitoxantrone | 9 | 22 | 31 | | | |
| | Bleomycin/MMC[d] | 2 | 13 | 15 | | | |
| | Epirubicin/MMC[d] | 10 | 23 | 33 | 14.1 | 3 | 0.003 |
| Stage | I/II | 7 | 2 | 9 | | | |
| | III/IV | | | | | | |
| | *III* | 20 | 51 | 71 | | | |
| | *IV* | 3 | 8 | 11 | 7.0[e] | 1 | 0.008 |
| | *Not known* | 1 | 0 | 1 | | | |
| Type | Serous | 18 | 34 | 52 | | | |
| | Clear | 4 | 11 | 15 | | | |
| | Unspecified | 7 | 6 | 13 | | | |
| | Other | | | | | | |
| | *Mucinous* | 1 | 7 | 8 | | | |
| | *Endometroid* | 0 | 1 | 1 | | | |
| | *Undifferentiated* | 1 | 2 | 3 | 4.3 | 3 | 0.23 |
| Differentiation | Poor | 12 | 24 | 36 | | | |
| | Moderate/well | | | | | | |
| | *Moderate* | 5 | 17 | 22 | | | |
| | *Well* | 3 | 3 | 6 | 0.02[e] | 1 | 0.89 |
| | Unspecified | 11 | 17 | 28 | | | |
| Best previous response | CR | 11 | 15 | 26 | | | |
| | PR | 8 | 15 | 23 | | | |
| | Static/progression | | | | | | |
| | *Static* | 1 | 6 | 7 | | | |
| | *Progression* | 1 | 22 | 23 | | | |
| | Adjuvant[f] | 10 | 3 | 13 | 21.6 | 3 | 0.0001 |
| Previous DDP | Yes | 28 | 55 | 83 | | | |
| | No | 3 | 6 | 9 | 0.0[e] | 1 | 1.0 |
| No. previous treatments | One | 26 | 48 | 74 | | | |
| | Two or more | | | | | | |
| | *2* | 4 | 9 | 13 | | | |
| | 3 | 1 | 4 | | 5 | 0.1 | 0.75 |

[a]Mann–Whitney U test; [b]Italicised sub-groups were pooled for calculation of $\chi^2$; [c]MD = mitoxantrone/*cis*-platinum; CP/ABC = *cis*-platinum/cyclophosphamide alternating with adriamycin/bleomycin/chlorambucil; [d]MMC = mitomycin C; [e]After Yate's correction; [f]Patients with no evaluable disease after primary surgery.

**Table II** Variables used in discriminant function analysis

| *Variable* | *Forms tested in discriminant analysis* |
|---|---|
| Age | Age; age$^2$; square root (age) |
| Interval | Interval; square root (interval); log10 (interval); ln(interval) |
| | Alternative single cutpoints (of 12, 14, 16, 18, 20 months) |
| | One variable with multiple cutpoints (1, 3, 6, 12, 18, 24 months) |
| Stage | Stage I/II *vs.* III/IV |
| Histology | Serous *vs.* rest; clear *vs.* rest; unspecified *vs.* rest |
| Differentiation | Poor *vs.* rest |
| Number of previous treatments | One *vs.* rest |
| Best previous response | CR/PR/Adjuvant *vs.* static/progression |
| Previous platinum chemotherapy | No *vs.* yes |
| Type of drugs in phase II study | Platinum *vs.* rest; mitoxantrone *vs.* rest |

improve the discrimination. When FIGO stage was entered, none of the remaining variables could improve discrimination between responders and non-responders.

The weights for interval and stage and constants derived from the analysis were used to calculate the 'classification scores' ($S_1$ and $S_2$) for each patient:

$S_1$ = score for patient classification into responder group $= -7.9 + 8.9 \times \text{stage value} + 1.7 \times \text{interval value}$

and

$S_2$ = score for patient classification into non-responder group $= -6.9 + 11.6 \times \text{stage value} + 0.6 \times \text{interval value}$

where the stage value for patients with FIGO stage I or II at presentation is 0 and with FIGO stage III or IV is 1; and the interval is the square root of the interval from the end of prior therapy to entering the phase II study in months.

Each of the patients was classified into the group for which they had the highest score (Table III). The function correctly classified 77.4% of patients who responded to chemotherapy and 88.5% of those who did not, with 84.8% being correctly classified overall.

Interval was by far the most important variable, and although FIGO stage gave a statistically significant improvement in discrimination between responders and non-responders, it may not usefully improve the percentage

**Table III** Patient classifiction using scores S1 and S2

| Classification | Clinical observation | |
|---|---|---|
| | Responded | Did not respond |
| Responder | 24 | 7 |
| Non-responder | 7 | 54 |
| Predictive value of test (%) | 77.4 | 88.5 |

**Table IV** Response rate using interval from previous treatment to phase II therapy only

| Interval (months) | Total no. | No. responding | % responding |
|---|---|---|---|
| <3 | 39 | 4 | 10 |
| 4–6 | 11 | 1 | 9 |
| 7–9 | 11 | 4 | 36 |
| 10–12 | 6 | 1 | 17 |
| 13–15 | 4 | 2 | 50 |
| 16–18 | 4 | 3 | 75 |
| 19–21 | 1 | 1 | 100 |
| >21 | 16 | 15 | 94 |

**Table V** Classification of patients using alternative single interval cutpoints

| Cutpoint (months) | % correctly classified | | |
|---|---|---|---|
| | Responders | Progressors | Overall |
| 3 | 87.1 | 57.4 | 67.4 |
| 6 | 83.9 | 73.8 | 77.2 |
| 9 | 71.0 | 85.2 | 80.4 |
| 12 | 67.7 | 93.4 | 84.8 |
| 15 | 61.3 | 96.7 | 84.8 |
| 18 | 51.6 | 98.4 | 82.6 |
| 21 | 48.4 | 98.4 | 81.5 |

classified correctly. Therefore, the value of interval alone was explored. A detailed breakdown of the relationship between interval and response is given in Table IV. Although the numbers in the sub-groups are small, these data suggest that patients progressing on primary treatment, or relapsing within 3–6 months of primary treatment have only a small chance of responding. Patients with a remission duration of greater than 15 months, however, stood a greater than 75% chance of responding to treatment.

The effect of using interval alone on the ability to predict responders and non-responders correctly is shown in Table V, where the results for a range of cutpoints are given. Adopting earlier cutpoints gives a better prediction of responders at the expense of more progressing patients being incorrectly classified as responders. When the latest cutpoint (i.e. 21 months) is adopted, virtually all the progressors are correctly identified at the expense of classifying responders incorrectly as non-responders. A cutpoint of 12–15 months gives the best overall classification of patients into responders and non-responders. Using this cutpoint for interval alone, the percentage of patients correctly classified is as good as that achieved by the discriminant function.

### Discussion

The evaluation of drugs for ovarian cancer in a phase II setting is difficult. The undoubted efficacy of established chemotherapy agents means that patients will be treated in phase II studies when they have progressed or relapsed. In addition, the patient population will be heterogeneous showing a wide range of characteristics. The studies in this analysis were chosen because the drugs had either shown previous activity or because of evidence of *in vitro* activity. This analysis demonstrates that there are other factors which are of importance in determining whether a patient will respond in a phase II study.

In the univariate analysis, interval between previous therapy and entry to the phase II study was significant, as was the presenting stage of the patient, the second line treatment chosen and the best previous response to therapy. In the discriminant analysis, however, only two factors were shown to be of importance, interval and the FIGO stage of the patient. Using these two variables, the discriminant analysis predicted 89% of those who did not respond and 75% of those who did, giving a correct prediction for 85% of patients overall. The importance of interval is emphasised by the observation that the response rate of those patients who progressed on primary treatment and received phase II therapy within 6 months of completing primary treatment was very low (5/50 = 10%) (Table IV). Correspondingly, those who had an interval of greater than 21 months between previous therapy and phase II treatment had a high response rate (19/21 = 90%). Indeed, in this study, it was possible to classify correctly 85% of patients by using an interval of 15 months or greater to predict those patients who would respond. The cutpoint of 15 months correctly predicted 61% of those who responded and 97% of those who did not.

While this is a small study which needs replicating, two of its findings are clear. Firstly, not all patients have the same probability of responding to phase II treatments. Secondly, even if future studies are able to improve the prediction of response, interval between previous treatment and entry to phase II treatment is likely to remain of importance. These observations have implications for the design of new phase II studies and also for the clinical management of patients with ovarian cancer relapsing from primary therapy.

The finding that not all patients have the same probability of responding in phase II study leads to two problems:

1. The response rate achieved in a particular study will depend not only on the efficacy of the agent being used, but also on the proportion of patients entered who have a very low probability of response.
2. The assumptions underlying the methods used to determine the required sample size for a phase II study may be violated. Two studies with the same number of patients would have a different chance of detecting an active new agent if they had a different proportion of patients with a low probability of response. This means that phase II studies may be failing to detect active new agents.

The entry criteria for phase II studies of new agents which are likely to have activity similar to existing agents, should exclude patients who have little chance of response. In ovarian cancer, this could be achieved by excluding patients who had progressed within 15 months of their previous therapy or more simply by not considering patients for phase II studies who have failed primary therapy, or who relapse within a few months. If it was thought that an agent was genuinely novel then this very poor group of patients might be used to identify agents with completely different activity.

These observations relate primarily to the evaluation of phase II treatments. The decision whether to treat a particular patient who has relapsed after primary therapy for ovarian cancer must remain with the clinician. The observations of this study, however, give some indication of the probability of response and may also influence the kind of treatment given.

We would like to thank all the clinicians who contributed to these studies, and also the West Midlands Cancer Research Campaign Trials Unit for providing the facilities for data collection and analysis. F.G.L., C.W.E.R. and K.K. were funded by the Cancer Research Campaign.

### References

HILGERS, R., RIVKIN, S., VON HOFF, D. & 5 others (1984). Mitoxantrone in epithelial carcinoma of the ovary. *Am. J. Clin. Oncol.*, **7**, 499.

KATZ, M., SCHWARTZ, P., KAPP, D. & 5 others (1981). Epithelial carcinoma of the ovary: current strategies. *Ann. Intern. Med.*, **95**, 98.

LAWTON, F.G., BLACKLEDGE, G. & MOULD, J.J. (1985). Alternating platinum combination chemotherapy in gynaecological malignancies. *Br. J. Cancer.*, **52**, 450.

LAWTON, F.G., PERREN, T.J., LUESLEY, D.M. & 4 others (1986). Combination bleomycin/mitomycin-C after failed cisplatinum and alkylating agent therapy in epithelial ovarian cancer. *Cancer Treat. Rep.*, **70**, 525.

LAWTON, F., BLACKLEDGE, G., MOULD, J.J., LATIEF, T., WATSON, R. & CHETIYAWARDANA, A. (1987). Phase II study of mitoxantrone (NSC-301739) in epithelial ovarian cancer. *Cancer Treat. Rep.*, **71**, 627.

LAWTON, F., BLACKLEDGE, G., REDMAN, C., LUESLEY, D. & MOULD, J. (1987). Mitoxantrone and cisplatinum in ovarian cancer: an overview. *Semin. Oncol.*, **12**, suppl. 4, 47.
studies. In *I Tumori Dell'Ovaio*, Batteli, T., Bonadonna, G., Maocchi, P., Mariuzzi, G.M., Mattioli, R. & Romanini, C. (eds) p. 397. Moduzzi.

MUSS, H.B., ASBURY, R., BUNDY, B. & 4 others (1984). Mitoxantrone (NSC 301739) in patients with advanced ovarian cancr: a phase II study of the Gynaecologic Oncology Group. *Am. J. Clin. Oncol.*, **7**, 737.

NEIJT, J., TEN BOKKEL HUININK, W., VAN DER BURG, M. & VAN OOSTEROM, A. (1986). Complete remission at laparotomy: still a gold standard in ovarian cancer? *Lancet*, **i**, 1028.

OZOLS, R.F. & YOUNG, R.C. (1984). Chemotherapy of ovarian cancer. *Semin. Oncol.*, **11**, 251.

REDMAN, C., LAWTON, F., STUART, N. & 7 others (1988). Phase II study of combination 4'epidoxorubicin and mitomycin C in recurrent epithelial ovarian cancer. *Cancer Chemother. Pharmacol.* (in the press).

THIGNPEN, T. & BLESSING, J. (1985). Current therapy of ovarian cancer: An overview. *Semin. Oncol.*, **12**, suppl. 4, 47.